# Supplementary material for: Evidence for Angiotensin II as a Naturally Existing Suppressor for the Guanylyl Cyclase A Receptor and Cyclic GMP Generation
Source: Int J Mol Sci. 2023 May 10;24(10):8547. doi: 10.3390/ijms24108547 (PMC10218449; doi:10.3390/ijms24108547)
Supplement: Supplementary file 1 [file ijms-24-08547-s001.zip › ijms-2366317-supplementary.pdf]

Evidence for Angiotensin II as a Naturally Existing Suppressor for the Guanylyl Cyclase A Receptor and Cyclic GMP Generation

(Supplemental Material)

Xiao Ma[1], Seethalakshmi R. Iyer[1], Xiaoyu Ma[1], Shawn H. Reginauld[1], Yang Chen[1], Shuchong Pan[1], Ye Zheng[1], Dante G. Moroni[1], Yue Yu[2], Lianwen Zhang[3], Valentina Cannone[1,4], Horng H. Chen[1], Carlos M. Ferrario[5], S. Jeson Sangaralingham[1,6], John C. Burnett, Jr.[1,6]

[1]Cardiorenal Research Laboratory, Department of Cardiovascular Medicine, Mayo Clinic, Rochester, MN, USA, [2]Department of Quantitative Health Sciences, Mayo Clinic, Rochester, MN, USA, [3]Department of Molecular Medicine, Mayo Clinic, Rochester, MN, USA, [4]Department of Medicine and Surgery, University of Parma, Parma, Italy, [5]Department of Surgery, Wake Forest School of Medicine, Winston Salem, NC, USA, [6]Department of Physiology and Biomedical Engineering, Mayo Clinic, Rochester, MN, USA.

Running title: ANGII as a Natural Suppressor of the GC-A/cGMP Pathway

Corresponding Author:

Xiao Ma, PhD

Cardiorenal Research Laboratory

Department of Cardiovascular Medicine

Mayo Clinic

200 First Street SW, Rochester, MN 55905

Phone: (+1)507-293-0242; Fax: (+1)507-266-4710

Emails: Ma.Xiao@mayo.edu

Supplementary Table S1. Baseline Characteristics of Healthy Subjects Subgrouped by Sex

Characteristics	Female Subjects (N=91)	Male Subjects (N=37)	*p* value
Age, years	58 ± 11	55 ± 15	0.26
Sex, female	100%	0%	---
BMI, kg/m^2	26 ± 5	27 ± 4	0.22
eGFR, $mL/min/1.73m^2$	84 ± 13	86 ± 13	0.36
**Plasma Variables**			
ANP, pg/mL	4.0 (4.0, 8.0)	4.0 (4.0, 4.0)	0.52
BNP, pg/mL	28.0 (16.3, 40.8)	19.0 (12.0, 27.0)	0.20
CNP, pg/mL	11.0 (9.1, 14.5)	13.3 (11.2, 15.6)	0.73
cGMP, pmol/mL	1.6 (1.2, 2,2)	1.8 (1.3, 2.9)	0.20
ANGII, pg/mL	4.4 (3.1, 6.1)	4.7 (3.7, 6.1)	0.54
Renin, ng/mL/hour	0.84 (0.54, 1.92)	1.45 (0.50, 2.18)	0.79
Aldosterone, ng/dL	6.1 (3.9, 11.4)	6.6 (3.9, 11.4)	0.36

Values are presented as mean ± SD, n (%), or median (interquartile range); other abbreviations as in **Table 1**.

Supplementary Table S2. Univariable Analysis of Plasma ANGII in Healthy Subjects

**N = 128**	**Log_{10}(Plasma ANGII)**		
**Predictor Variable**	**β (SE)**	**r**	***p* value**
Age	-0.003 (0.002)	-0.13	0.15
Sex, female	-0.02 (0.03)	-0.03	0.58
BMI	0.0004 (0.0005)	0.002	0.94
eGFR	0.001 (0.002)	0.07	0.43
Log_{10}(Plasma ANP) #	-0.13 (0.09)	-0.24	0.12
Log_{10}(Plasma BNP)	-0.14 (0.08)	-0.16	0.07
Log_{10}(Plasma CNP)	-0.03 (0.13)	-0.02	0.81
Log_{10}(Plasma cGMP)	-0.18 (0.10)	-0.14	0.11

Log10 transformation was performed on plasma ANP, BNP, CNP, cGMP, and ANGII for approximate normality of distribution.

#N = 44 as subjects with plasma ANP at or below the minimal detection level (4 pg/mL) were excluded for this analysis.

SE, standard error; r, Pearson correlation coefficient; other abbreviations as in **Table 1**.

Supplementary Figure S1. Correlations between cGMP and CNP in Healthy Subjects Grouped by ANGII Levels.

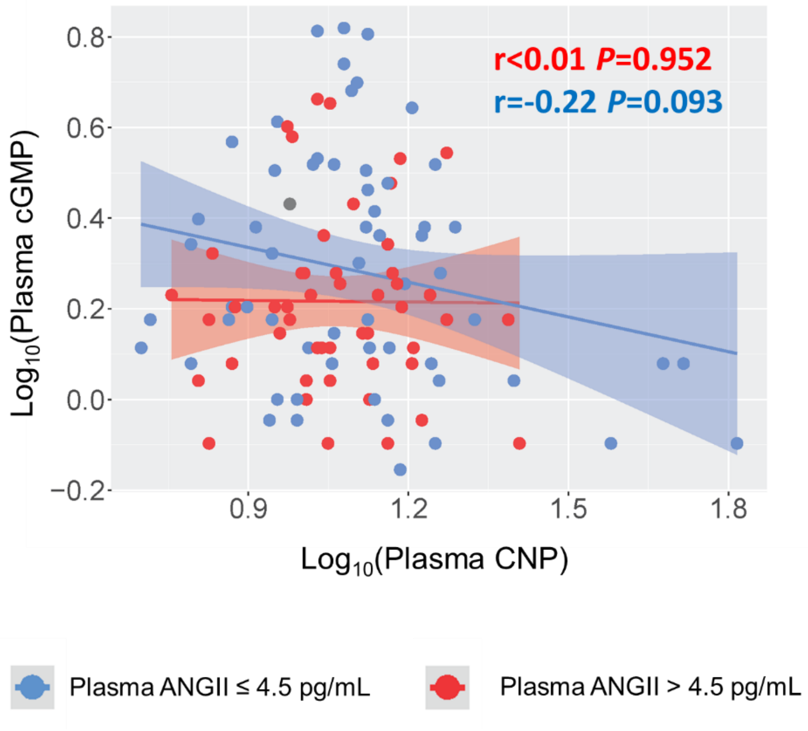

Supplementary Figure S2. ANGII Effect on Endogenous cGMP In Vivo.

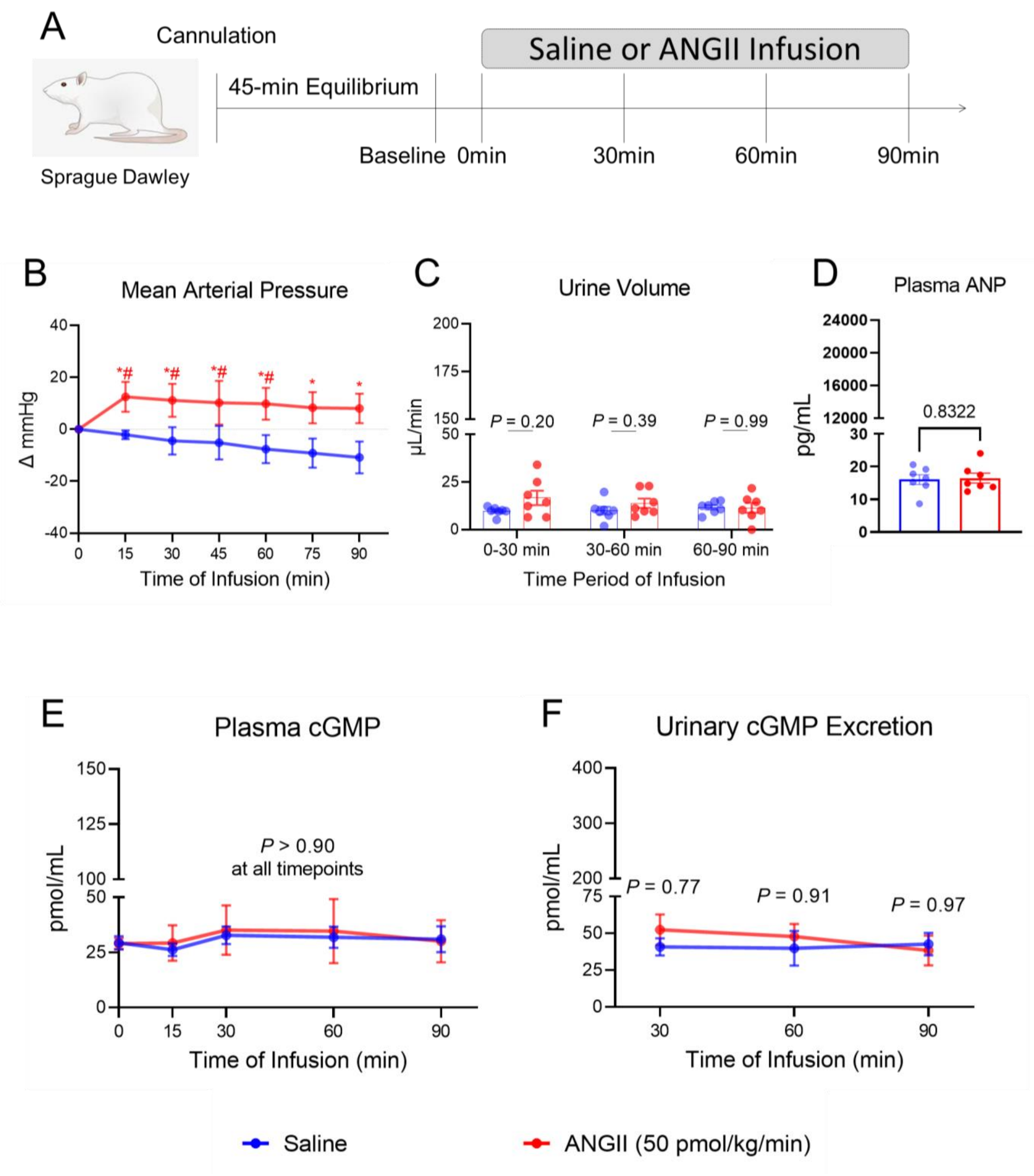

Supplementary Figure S3. In Vitro cGMP Generation in HEK293 Transfected Cells.

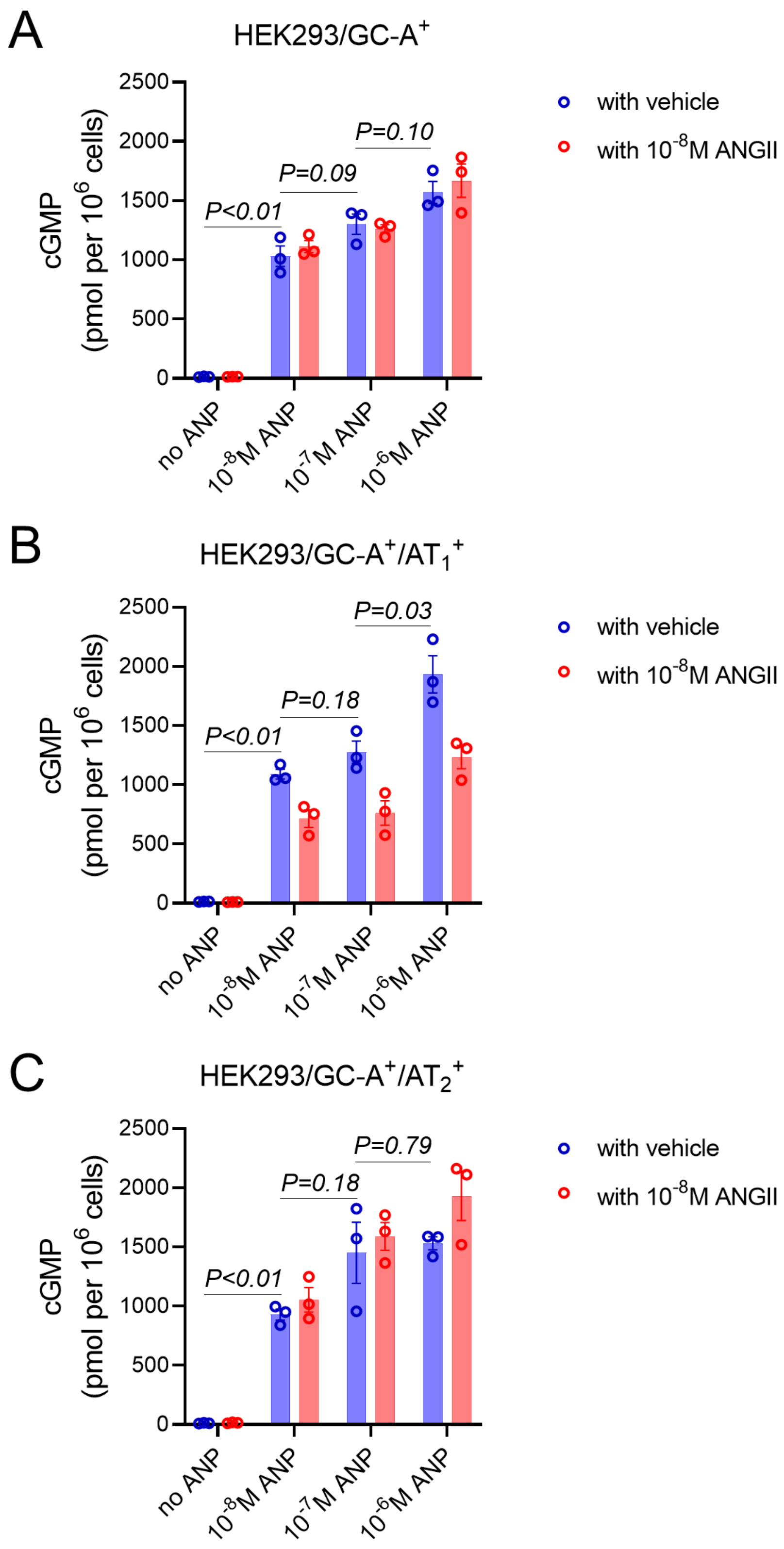

Supplementary Figure S4. In Vitro cGMP Generation in Human Renal Proximal Tubular Epithelial Cells.

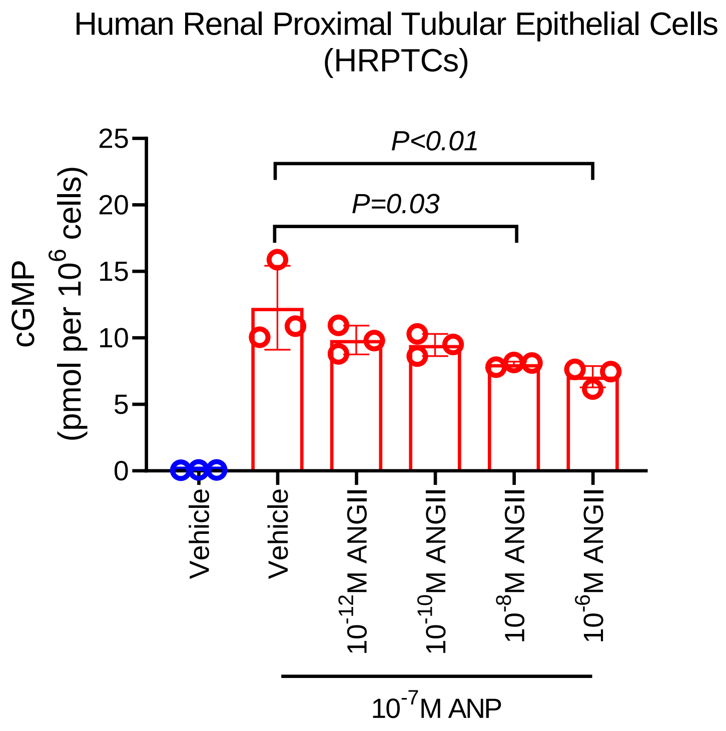

Supplementary Figure S1. Correlations between cGMP and CNP in Healthy Subjects Grouped by ANGII Levels. Linear correlation between plasma CNP and plasma cGMP. Each dot represents one subject. Blue indicates the subjects with plasma ANGII ≤ 4.5 pg/mL; Red indicates the subjects with plasma ANGII > 4.5 pg/mL. Pearson correlation coefficient r and *P* value were shown for each constructed linear regression model.

Supplementary Figure S2. ANGII Effect on Endogenous cGMP In Vivo. (A) The acute protocol study design to evaluate the effect of ANGII infusion in normal rats. (B) Mean arterial pressure. * indicates $P<0.05$ compared to saline group; # indicates $P<0.05$ compared to corresponding baseline. (C) Urinary output represented by urine volume collected at every 30-min interval during continuous infusion. (D) Plasma ANP at 90 mins after saline or ANGII infusion. (E) Plasma cGMP, no statistical difference was found between two groups or compared to baseline at all timepoints. (F) Urinary cGMP excretion. no statistical difference was found between two groups or compared to baseline at all timepoints. N=7 in saline group, N=7 in ANGII group.

Supplementary Figure S3. In Vitro cGMP Generation in HEK293 Transfected Cells. Absolute values of cGMP generation in different HEK293 transfected cells in response to ANP with or without ANGII (10^{-8} M). The effect of vehicle alone or ANGII alone in corresponding HEK293 transfected cells were labeled as “no ANP” as negative control. Values of cGMP were quantified in pmol per 10^{6} cells. Each dot represents one biological replicate. Shown *P* values are comparisons between different doses of ANP without ANGII (blue bars) using two-side unpaired t test.

Supplementary Figure S4. In Vitro cGMP Generation in Human Renal Proximal Tubular Epithelial Cells.

Absolute values of cGMP generation in human renal proximal tubular epithelial cells (HRPTCs) in response to ANP (10^{-7}M) with or without different concentrations of ANGII (10^{-12} to 10^{-6} M). Values of cGMP were quantified in pmol per 10^{6} cells. Each dot represents one biological replicate. Only significant *P* values (<0.05) are shown in comparisons among ANP-treated groups using one-way ANOVA following by multiple comparison.
